# Prior COVID-19 Immunization Does Not Cause IgA- or IgG-Dependent Enhancement of SARS-CoV-2 Infection

**DOI:** 10.3390/vaccines11040773

**Published:** 2023-03-31

**Authors:** Melyssa Yaugel-Novoa, Blandine Noailly, Fabienne Jospin, Anne-Emmanuelle Berger, Louis Waeckel, Elisabeth Botelho-Nevers, Stéphanie Longet, Thomas Bourlet, Stéphane Paul

**Affiliations:** 1 CIRI—Centre International de Recherche en Infectiologie, Team GIMAP, Université Claude Bernard Lyon 1, Inserm, U1111, CNRS, UMR530, F42023 Saint-Etienne, France; 2 Immunology Department, University Hospital of Saint-Etienne, F42055 Saint-Etienne, France; 3 Infectious Diseases Department, University Hospital of Saint-Etienne, F42055 Saint-Etienne, France; 4 Infectious Agents and Hygiene Department, University Hospital of Saint-Etienne, F42055 Saint-Etienne, France; 5 CIC 1408 Inserm Vaccinology, University Hospital of Saint-Etienne, F42055 Saint-Etienne, France

**Keywords:** SARS-CoV-2, ADE, IgA, Delta, Omicron, vaccines

## Abstract

Antibody-dependent enhancement (ADE) can increase the rates and severity of infection with various viruses, including coronaviruses, such as MERS. Some in vitro studies on COVID-19 have suggested that prior immunization enhances SARS-CoV-2 infection, but preclinical and clinical studies have demonstrated the contrary. We studied a cohort of COVID-19 patients and a cohort of vaccinated individuals with a heterologous (Moderna/Pfizer) or homologous (Pfizer/Pfizer) vaccination scheme. The dependence on IgG or IgA of ADE of infection was evaluated on the serum samples from these subjects (twenty-six vaccinated individuals and twenty-one PCR-positive SARS-CoV-2-infected patients) using an in vitro model with CD16- or CD89-expressing cells and the Delta (B.1.617.2 lineage) and Omicron (B.1.1.529 lineage) variants of SARS-CoV-2. Sera from COVID-19 patients did not show ADE of infection with any of the tested viral variants. Some serum samples from vaccinated individuals displayed a mild IgA-ADE effect with Omicron after the second dose of the vaccine, but this effect was abolished after the completion of the full vaccination scheme. In this study, FcγRIIIa- and FcαRI-dependent ADE of SARS-CoV-2 infection after prior immunization, which might increase the risk of severe disease in a second natural infection, was not observed.

## 1. Introduction

Antibody-dependent enhancement (ADE) is known to increase infection and severe disease rates due to various viruses, including coronaviruses, such as MERS (Middle East respiratory syndrome). Two types of ADE have been described: intrinsic and extrinsic ADE. The first one refers to the cellular mechanisms that increase viral replication and release of new virions, while the second one refers to the elements relative to virus–receptor interaction and viral cell entry [1]. The possibility of ADE in COVID-19 (Coronavirus disease) has been a matter of debate. Some studies show an ADE effect in vitro (refs. [2,3,4,5,6,7]), but clinical and epidemiological studies show that prior immunization does not increase the likelihood of severe disease [7,8,9,10,11,12,13]. The ADE of SARS-CoV-2 (severe acute respiratory syndrome coronavirus 2) infection has been reported to occur via various routes in vitro. For example, sera from patients convalescing from COVID-19 can enhance infection via the FcγR or C1q protein [2,3,4]. Furthermore, sera from convalescent individuals, but not from vaccinated individuals, has been reported to enhance virus uptake by monocytes in vitro without the production of viral particles. However, pyroptosis is triggered, which can increase inflammation in COVID-19 [14]. The antibodies responsible for ADE have been reported to target epitopes different from those targeted by antibodies capable of fully neutralizing infection [5,6,7,15]. The NTD (N-terminal domain) region of the spike protein is one of the ADE epitopes identified, with monoclonal antibodies targeting this region reported to increase SARS-CoV-2 infection in vitro in ACE2 (angiotensin-converting enzyme 2)-overexpressing cells [5,15]. However, other in vitro studies with sera from patients convalescing from COVID-19 have found no evidence of ADE, even at subneutralizing concentrations [16,17].

We investigated the possible ADE effect of sera from vaccinated individuals and from SARS-CoV-2-infected patients with severe or asymptomatic COVID-19 following challenges with the Delta (B.1.617.2 lineage) or Omicron (B.1.1.529 lineage) variants. We investigated whether ADE of SARS-CoV-2 infection occurred, and its possible dependence on IgG or IgA, using an in vitro model based on HEK-293 cells expressing the CD16 and CD89 receptors, respectively.

## 2. Materials and Methods

### 2.1. Cohorts

Twenty-six individuals who had never had COVID-19 and had been vaccinated with the Pfizer BNT162b2 and/or Moderna mRNA-1273 vaccines were included in a prospective longitudinal cohort study conducted at CHU Nord de Saint-Etienne (Saint-Etienne, France). Blood was collected before and two weeks after administration of the booster dose of vaccine. We also studied a cohort of twenty-one PCR-positive SARS-CoV-2-infected patients with the B.1 strain. COVID-19 patients were split into two groups according to disease severity: 10 patients with mild or asymptomatic disease and 11 patients with severe disease requiring admission to the ICU. Serum samples were collected between 14 and 30 days post-symptom onset (PSO). Written informed consent for participation was obtained from all subjects; ethics approval was obtained from the CPP Ile de France V (NCT04648709).

### 2.2. Cell Models

HEK-CD89+ (FcαRI), HEK-CD16+ (FcγRIIIa), and HEKWT cell lines were obtained from InvivoGen and previously described [18]. All cells were maintained in Dulbecco′s modified Eagle medium (DMEM) supplemented with 10% SVF and 1% antibiotic-antimycotic (AAT). Cells were harvested with trypsin/EDTA solution and the stable expression of FcR was checked by flow cytometry before each experiment. All cell lines were maintained at 37 °C, in a humidified atmosphere containing 5% CO_2_.

### 2.3. In Vitro Assay for Antibody-Dependent Enhancement (ADE)

The in vitro ADE assay was performed with cell lines expressing FcR (HEK-CD16+ or HEK-CD89+). Serum samples from vaccinated individuals and COVID-19 patients (1:100 dilution) were incubated with 0.7 MOI of Delta (B.1.617.2 lineage) or Omicron (B.1.1.529 lineage) variants for 1 h at 37 °C. HEK-CD16+, HEK-CD89+ or HEKWT cells were incubated with the mixture of serum plus virus for 120 h at 37 °C. Viral amplification was assessed by RT-PCR on the culture supernatant. We then calculated and compared the ratios of the amount of virus in each sample to that in a viral control without serum. Two monoclonal antibodies were used as positive controls: an anti-RBD IgG (Ref: G_SAR2-RBD_X30F12-PU; BCell Design, Limoges, France) was used for the CD16 test, and an anti-S IgA (Ref: MAB12439-100; Native Antigens, Kidlington, United Kingdom) was used for the CD89 test. Eight human serum samples from 2005 were used as the negative control, and the mean value for these samples plus twice the standard deviation was used as the cut-off for enhancement.

### 2.4. SARS-CoV-2 RT-PCR for Viral Amplification

SARS-CoV-2 in the culture supernatant was quantified by RT-PCR without nucleic acid extraction, with the Luna Universal Probe One-Step RT-qPCR Kit (Ref. E3006L, New England Biolabs, Ipswich, MA, USA) [19]. Briefly, 5 μL of supernatant was diluted 1/10 with Dnase-free and Rnase-free water and mixed with the reaction solution to obtain a total volume of 14 μL. The reaction solution contained 5 μL Luna^®^ Universal Probe One-Step Reaction Mix, 0.5 μL Luna^®^ WarmStart^®^ RT Enzyme Mix and 1.5 μL of a mixture of primers at 400 nM (E_Sarbeco_F: ACAGGTACGTTAATAGTTAATAGCGT and E_Sarbeco_R: ATATTGCAGCAGTACGCACACA), and the probe at a concentration of 200 nM (E_Sarbeco_P1: FAM-ACACTAGCCATCCTTACTGCGCTTCG-BBQ) [20]. The RT-PCR was initialized with a reverse transcription step at 55 °C for 10 min, followed by 40 cycles of denaturation at 95 °C for 10 s and annealing at 60 °C for 60 s. A viral standard curve was obtained for each analysis.

### 2.5. Live Virus Neutralization Experiments

Virus neutralization was performed as previously described [21]. Briefly, serial 2-fold dilutions (tested in duplicate, starting at 1:10) of the serum specimens in culture medium were mixed at equal volume with the live SARS-CoV2 virus. After incubation of the mix for 30 min at room temperature, 150 µL of the mix was transferred into 96-well microplates covered with a monolayer of Vero E6 cells to achieve a viral concentration of 100 TCID50/well. The plates were incubated at 37 °C in a 5% CO_2_ atmosphere for 5 days. After this time, the cytopathic effect was evaluated, and neutralization was recorded if less than 50% of the cells were damaged. The neutralizing titer was expressed as the inverse of the higher serum dilution that exhibited neutralizing activity; a threshold of 20 was used (PRNT50 titer ≥ 20). All experiments were performed in a biosafety level 3 laboratory. The different viral strains that were used were sequenced and deposited on GISAID (GISAID accession numbers: EPI_ISL_1707038, 19A (B.38); EPI_ISL_1904989, Delta (B.1.617.2); and EPI_ISL_7608613, Omicron (B.1.1.529)).

### 2.6. Statistical Analysis

The statistical significance of the difference between groups for each set of conditions was determined by one-way ANOVA with Tukey’s test for multiple comparisons. Statistical significance was defined as *p* < 0.05. All statistical calculations were performed and graphs were generated with GraphPad Prism 9.5.0 (GraphPad, San Diego, CA, USA).

## 3. Results

Prior Immunization Does Not Enhance SARS-CoV-2 Infection

We investigated whether prior immunization could enhance a new SARS-CoV-2 infection by studying two cohorts: a cohort of patients infected with SARS-CoV-2 (B.1 lineage) and a cohort of individuals who had been vaccinated with either a heterologous (Moderna mRNA1273/Pfizer BNT162b2) or homologous (Pfizer BNT162b2/Pfizer BNT162b2) vaccination scheme. The characteristics of the study population are shown in Table 1.

**Figure 1:**
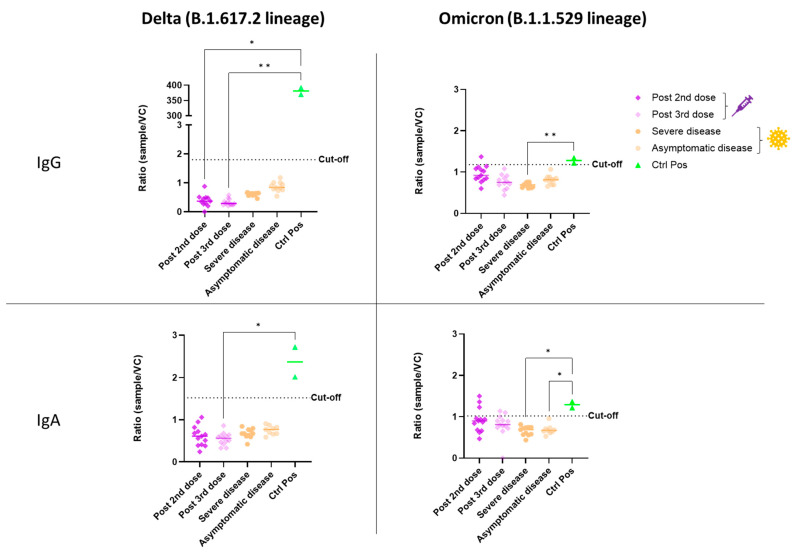
In vitro ADE of SARS-CoV-2 infection from vaccinated (syringe symbol) or COVID-19 patients (virus symbol) against Delta and Omicron variants. The ADE effect was evaluated using HEK-293 CD16+ or CD89+ cells. Each sample was tested simultaneously in both models (CD16 and CD89) and against both viral variants. The amount of virus present in the supernatant was determined after five days of infection by a SARS-CoV-2-specific RT-PCR. The ratio of the amount of virus in each sample and that in a viral control without serum (sample/VC) is shown. Each dot corresponds to the mean of duplicate values from a single patient. An anti-RBD IgG was used as the positive control for the CD16 test, and an anti-S IgA was used for CD89. Sera were used at a 1:100 dilution. The cut-off was determined as the mean value for eight pre-pandemic serum samples plus two standard deviations. The statistical significance of differences between groups in a Kruskal–Wallis test with Dunn’s tests for multiple comparisons is shown for each set of conditions (* *p* < 0.05; ** *p* < 0.01).

**Figure 2:**
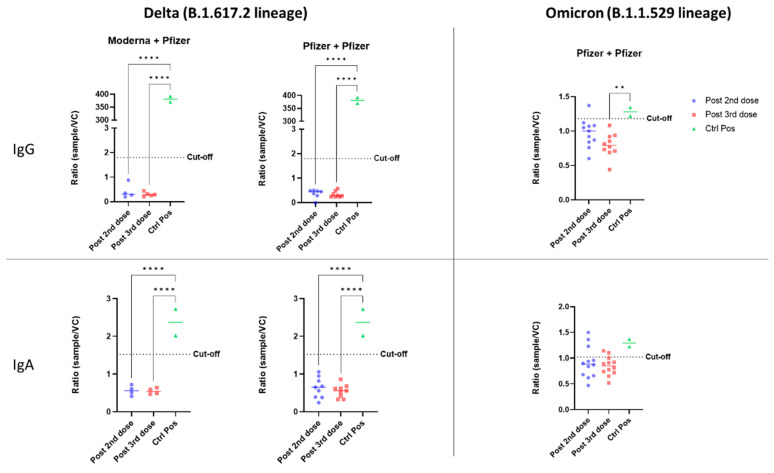
In vitro ADE of SARS-CoV-2 infection from individuals vaccinated with different vaccination schemes (heterologous: Moderna/Pfizer; or homologous: Pfizer/Pfizer). The ADE effect was evaluated with the Delta and Omicron variants, and the involvement of IgG and Ig A antibodies was assessed using HEK-293 CD16+ or CD89+ cells. Viral quantification was measured by a SARS-CoV-2-specific RT-PCR. The ratio of the amount of virus for each sample to that for a viral control without serum (sample/CV) is shown. Each dot corresponds to a single patient. An anti-RBD IgG was used as a positive control for the CD16 test, and an anti-S IgA was used for CD89. Sera were used at a 1:100 dilution. The cut-off was determined as the mean for eight pre-pandemic serum samples plus two standard deviations. The statistical significance of differences between groups in ANOVA with Tukey tests for multiple comparisons is shown for each set of conditions (** *p* < 0.01, **** *p* < 0.0001).

The ADE of infection was evaluated in vitro with either the Delta or Omicron variant of SARS-CoV-2. Our model consisted of HEK-293 cells expressing the FcR for either IgG (CD16) or IgA (CD89), to facilitate FcR-dependent infection. Firstly, HEK-293 cells without CD16 or CD89 expression were used to control non-FcR specific effects of sera (Figure S1 (Supplementary Materials)). None of the serum samples from vaccinated or SARS-CoV-2-infected individuals enhanced the infection of HEK-293 cells with the Delta variant via either the CD16 or CD89 receptor (Figure 1). For both homologous and heterologous vaccination schemes, negative results for ADE were obtained after both the second and booster dose (Figure 2).

**Figure 3:**
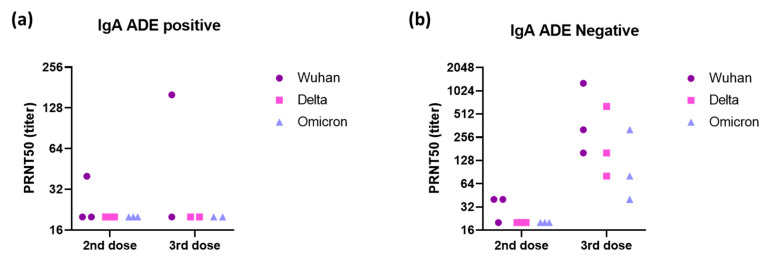
Neutralizing activity of (**a**) three positive and (**b**) three negative sera to IgA-dependent ADE against the Omicron variant (sera dilution starting at 1:20) was evaluated against Wuhan, Delta, and Omicron strains using a plaque reduction neutralization test (PRNT) with VeroE6 cells. Each dot corresponds to one patient.

In evaluations of ADE of infection with the Omicron variant, a positive signal was obtained for some vaccinated individuals relative to the eight pre-pandemic serum samples used to define the cutoff. However, sera from COVID-19 patients did not enhance infection in vitro (Figure 1). After the second dose of the homologous vaccination scheme, more serum samples tested positive for ADE of infection dependent on the CD89 receptor than for ADE of infection dependent on CD16, suggesting that IgA in the serum may enhance infection with the Omicron variant (Figure 2). Those patientswere as aged as the others and had positive levels of QUANTIFERON and anti-S IgG antibodies (Figure S2). However, they had no neutralizing antibodies against the Omicron variant (Figure 3). Nevertheless, after administration of the booster dose (a third administration of a half dose of mRNA vaccine), most serum samples tested negative for ADE (Figure 2). Those who were still positive for ADE did not develop neutralizing antibodies two weeks after the third dose of the vaccine (Figure 3). In addition, ADE IgG-dependence is negatively correlated with neutralizing titers and anti-SARS-CoV-2 antibody levels when facing the Delta variant (Figure S3a,b). However, ADE IgA-dependence was only negatively correlated with neutralizing activity and anti-SARS-CoV-2 antibody levels when facing the Omicron variant (Figure S3c,d). These results highlight the importance of a good neutralizing response to avoid the ADE phenomenon. There was no significant difference between doses, but there was a clear trend towards lower rates of ADE after the booster dose, emphasizing a decrease in the risk of possible disease aggravation due to immunity to a prior SARS-CoV-2 variant.

## 4. Discussion

ADE has been a major subject of concern in different viral diseases such as Dengue, Zika, and respiratory syncytial virus (RSV) [1,22]. It has been suggested that ADE may contribute to SARS-CoV2 infection in vitro, but we show here that infection is not enhanced after vaccination or prior natural infection. These results contrast with those of previous studies in which patients with severe disease or convalescent serum enhanced SARS-CoV-2 infection in vitro via FcγR [2,4,23]. For instance, Shimizu et al. showed that the serum of patients with severe disease patients increased viral infection as well as IL-6 production in a myeloid cell line, suggesting that serum antibodies can contribute to the cytokine storm present in COVID-19 disease [23]. In a more recent study, the same group reported ADE of infection in some monoclonal antibodies as well as in sera from vaccinated patients [24]. However, ADE was seen within a narrow window of antibody and serum concentrations. The time match between this antibody concentration and an effective viral quantity seems unlikely during natural infection. Furthermore, we must not forget that the cellular immune response is also playing a role against SARS-CoV-2 infection and that the ADE of infection seen in vitro might be subclinical during natural infection. On the other hand, our study is in line with other studies showing the absence of ADE in SARS-CoV-2 infection [16,17]. Clark et al. showed that neutralizing titers of convalescent plasma did not change in the presence of ACE2 and FcαR or FcγRIIA [16]. Garcia-Nicolas et al. did not find evidence for ADE of infection in human monocyte-derived macrophages (hMDM) or pro-inflammatory cytokine responses when evaluating convalescent serum [17].

The mild ADE effect observed in our study when sera from vaccinated individuals were incubated with the Omicron variant can be explained by the mutations in the Omicron spike protein relative to the strain used to produce the vaccine. However, we also found that after full vaccination (two full doses plus a half-dose booster), the ADE effect in vitro decreased substantially. This finding is consistent with the observation that, despite the lower neutralizing efficacy of antibodies against the Omicron variant, the number of patients with severe disease is not higher after vaccination [12,13]. Furthermore, preclinical and clinical studies have suggested that no ADE occurs in vivo [7]. For example, vaccination/challenge studies in non-human primates have reported no increase in disease severity in this animal model [8,9]. In addition, treatment with convalescent plasma did not increase the risk of severe disease in COVID-19 patients [10,11,25].

This study has some limitations. Even though CD16 is a known FcγR by its ADCC activity, notably in NK cells, it is not the only FcγR expressed in human immune cells. Therefore, results might be different if other FcγRs are tested. Nevertheless, as discussed earlier, some studies have tested the ADE of infection in vitro with other FcγRs and no ADE has been seen, for example, with the FcγRIIA [16]. In addition, the HEK-293 tested here were ACE2 negatives and it has been previously shown that the ADE of SARS-CoV-2 infection might require ACE2 as a co-receptor [3].

Overall, in this study, the FcγRIIIa- and FcαRI-dependent ADE of SARS-CoV-2 infection after prior immunization, which might increase the risk of severe disease in a second natural infection, was not observed.

## Figures and Tables

**Table 1:** Characteristics of the subjects studied, by group.

Characteristics	Groups		
	Severe Disease (*n* = 19)	Mild/Asymptomatic Disease (*n* = 36)	Vaccinated (*n* = 26)
M/F sex ratio	3.75	0.29	0.30
Age (years), median (IQR)	70 (61.5–71.5)	34 (29–40.3)	85 (29–86)

## Data Availability

Not applicable.

## Supplementary Materials

The following supporting information can be downloaded at: https://www.mdpi.com/article/10.3390/vaccines11040773/s1, Figure S1: Analysis of the ADE of SARS-CoV-2 infection in vitro with serum samples from individuals vaccinated or COVID-19-positive patients; Figure S2: Specific antibody response anti-SARS-CoV-2. Figure S3: Specific antibody response anti-SARS-CoV-2.
