# Supplementary material for: Prior COVID-19 Immunization Does Not Cause IgA- or IgG-Dependent Enhancement of SARS-CoV-2 Infection
Source: Vaccines (Basel). 2023 Mar 31;11(4):773. doi: 10.3390/vaccines11040773 (PMC10141984; doi:10.3390/vaccines11040773)
Supplement: Supplementary file 1 [file vaccines-11-00773-s001.zip › vaccines-2301928-supplementary.pdf]

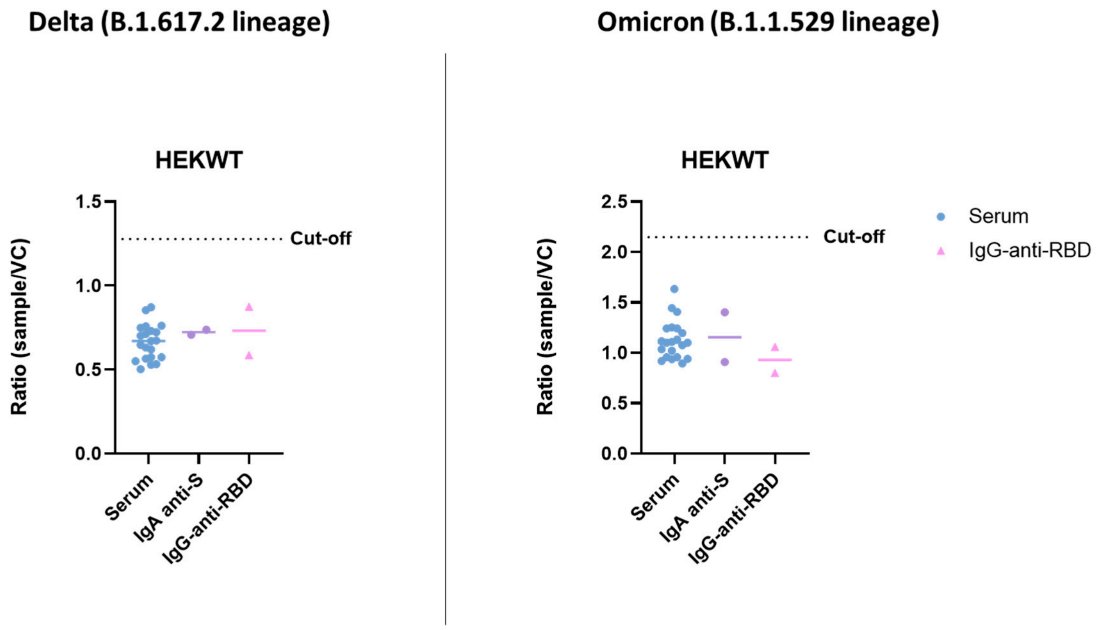

Figure S1. Analysis of the ADE of SARS-CoV-2 infection *in vitro* with serum samples from individuals vaccinated or COVID-19-positive patients. The anti-RBD IgG and anti-S IgA used as positive controls in the CD16/CD89 tests were also tested. The ADE effect was evaluated with HEK wild-type cells (HEKWT) against the delta and omicron variants. The ratio of the amount of virus for each sample to that for a viral control without serum (sample/VC) is shown. Each dot corresponds to a single patient. The cut-off was determined as the mean for eight pre-pandemic serum samples plus two standard deviations.

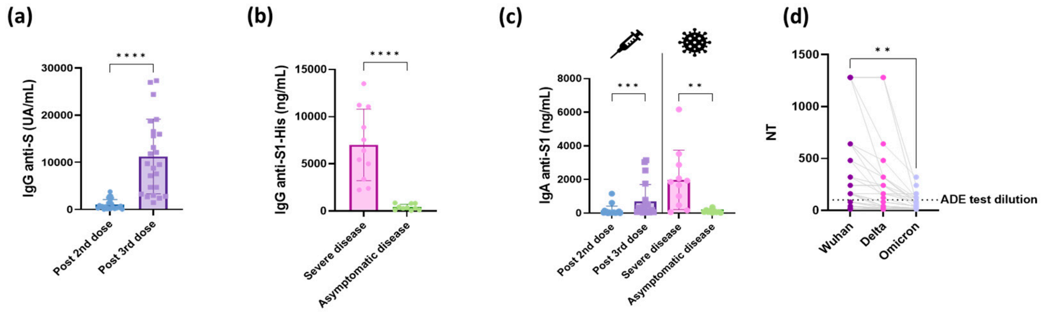

Figure S2. Specific antibody response anti-SARS-CoV-2. IgG **(a and b)** and IgA **(c)** against spike-1 (S1) were measured using ELISA in vaccinated individuals (designated with a syringe symbol) and COVID-19 patients (designated with a virus symbol). *P* value was calculated using an unpaired t-test. **(d)** Neutralizing titers (NT) for each tested serum against Wuhan, Delta, and Omicron variants were measured by a live-virus neutralization test. *P* value was calculated using a Kruskal-Wallis test with Dunn's tests for multiple comparisons. Each dot represents one individual. **(e-h)** Correlation matrices between ADE, viral neutralizing titers, and anti-SARS-CoV-

2 Ab concentrations. Correlation coefficients were calculated using Spearman's correlation tests and they were plotted using GraphPad Prism v.9.5.1. for panels A-C (*$P < 0.05$, **$P < 0.01$, ***$P < 0.001$, and ****$P < 0.0001$).

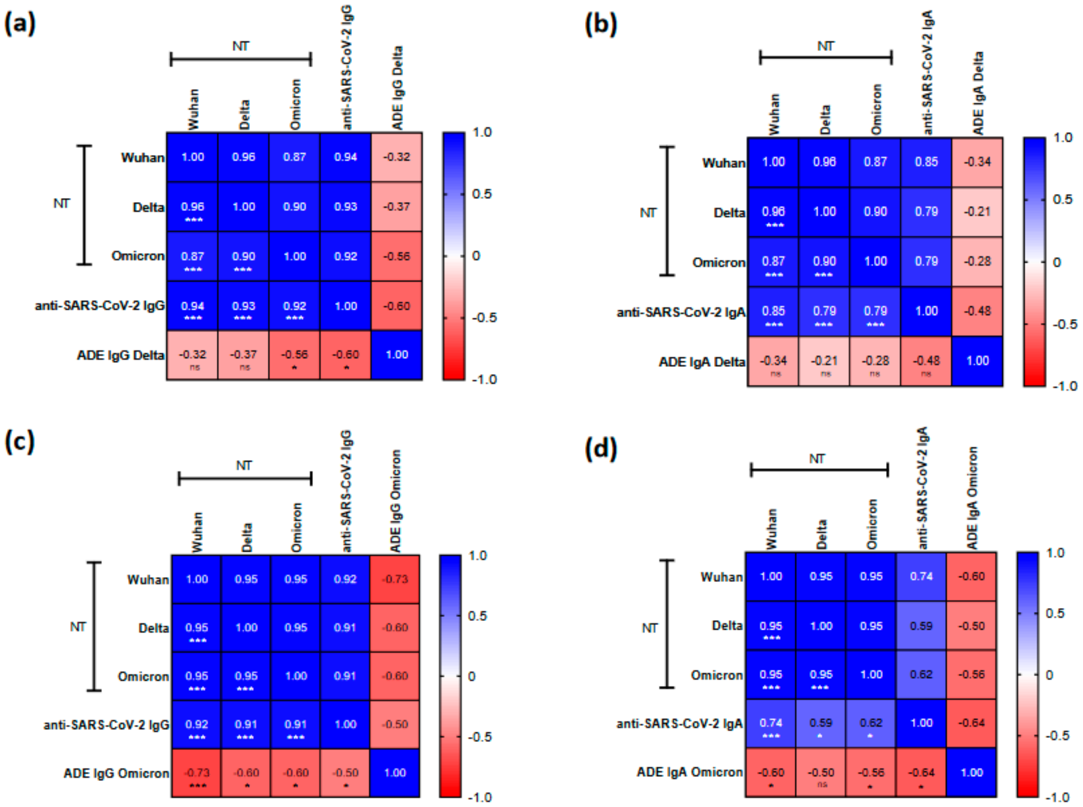

Figure S3. Specific antibody response anti-SARS-CoV-2.Each dot represents one individual. (a-d) Correlation matrices between ADE, viral neutralizing titers, and anti-SARS-CoV-2 Ab concentrations. Correlation coefficients were calculated using Spearman's correlation tests and they were plotted using GraphPad Prism v.9.5.1. for panels A-C.
